# Supplementary material for: ACEK Biosensor for the Minute-Scale Quantification of Breast Cancer ctDNA
Source: Sensors (Basel). 2024 Jan 15;24(2):547. doi: 10.3390/s24020547 (PMC10818266; doi:10.3390/s24020547)
Supplement: Supplementary file 1 [file sensors-24-00547-s001.zip › sensors-2801780-supplementary.pdf]

The Supporting Information for

ACEK Biosensor for the Minute-Scale Quantification of Breast Cancer ctDNA

Ke Wang [1], Xiaogang Lin [1,*], Maoxiao Zhang [1], Mengjie Yang [1], Xiang Shi [1], Mingna Xie [1] and Yang Luo [2,*]

[1] Key Laboratory of Optoelectronic Technology and Systems of Ministry of Education of China, Chongqing University, Chongqing 400044, China; kewang@stu.cqu.edu.cn (K.W.); 15223897701@163.com (M.Z.); 202208131095@stu.cqu.edu.cn (M.Y.); shixiangcqu98@163.com (X.S.); mingnaxie2017cqu@163.com (M.X.)

[2] Center of Smart Laboratory and Molecular Medicine, NHC Key Laboratory of Birth Defects and Reproductive Health, School of Medicine, Chongqing University, Chongqing 400044, China

* Correspondence: xglin@cqu.edu.cn (X.L.); luoy@cqu.edu.cn (Y.L.)

S.1. Electrochemical Responses at Different Frequencies

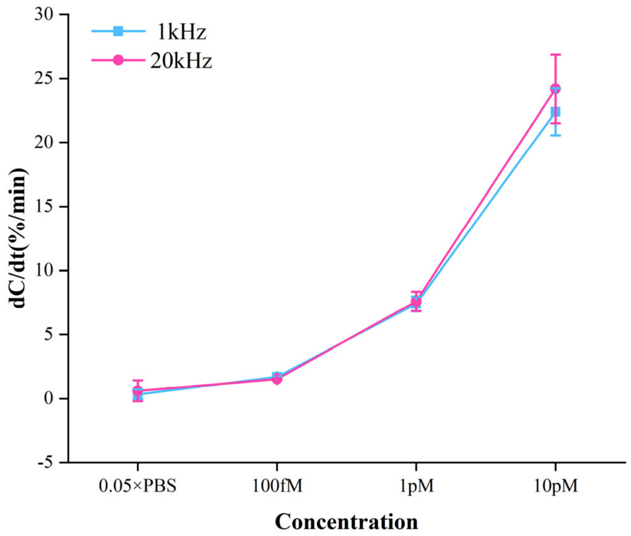

Figure S1. The rate of capacitance change of 100 fM, 1 pM, and 10 pM ctDNA solutions and 0.05× PBS was detected at 1 kHz and 20 kHz using the method of Au–S bonding immobilized probes.

The method of Au–S bonding immobilized probes was used as an example, a chip with a size of 5 μm × 5 μm was used, the AC signal voltage was set at 100 mV, and the frequencies were set to 1 kHz and 20 kHz. ctDNA solutions of 100fM, 1pM, and 10pM concentrations were tested, and 0.05 × PBS was used as a blank control. The test results are shown in Figure S1. The small differences in the test results for the 100 fM, 1 pM, and 10 pM ctDNA solutions at the two different frequencies indicate that the test results are relatively insensitive to frequency, and thus the ACET effect dominates the test procedure. In addition, the rate of capacitance change was more pronounced at 20 kHz; therefore, the applied AC signal frequency was set to 20 kHz for all subsequent experiments.

S.2. Electrochemical Responses at Different Chip Sizes

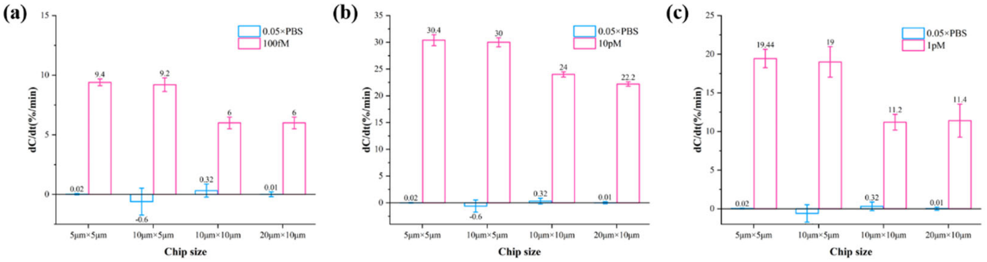

Figure S2. The rate of capacitance change of ctDNA solutions of (**a**) 100 fM, (**b**) 1 pM, and (**c**) 10 pM was detected using the method of Au–S bonding immobilized probes under four size chips.

By analyzing the conductivity of the background solution 0.05× PBS, it is known that the ACET effect dominates in this study. The microcurrent velocity of ACET is expressed as [1]:

$$u_{ACET} = 5\times10^{-4}\frac{\varepsilon_m \sigma V^4}{k\eta r}\left|\frac{1}{\sigma}\cdot\frac{\partial\sigma}{\partial T}\right| \quad (1)$$

where ε_m is the dielectric constant of the solution, σ is the conductivity of the solution, V is the magnitude of the voltage to which the AC signal is applied, k is the thermal conductivity of the solution, η is the viscosity of the solution, r is half of the electrode gap, and T is the absolute temperature.

From equation (1), the ACET microfluidic velocity is only affected by the size of the fork-finger electrode gap, independent of the width of the fork-finger electrode. Four chip sizes of 5 μm × 5 μm, 10 μm × 5 μm, 10 μm × 10 μm, and 20 μm × 10 μm were tested by the Au–S bond fixation probes method as an example. The frequency and voltage of the AC signal were set to 20 kHz and 300 mV. 100 fM, 1 pM, and 10 pM of ctDNA solution were tested using these four chip sizes, and 0.05×PBS was used as a blank control. The test results are shown in Figure S2. From the test results in Figure S2(a), Figure S2(b), and Figure S2(c), when detecting different concentrations of ctDNA solutions, the change of electrode width does not affect the normalized capacitance change rate when the electrode gap r is fixed. The smaller the electrode gap r, the larger the rate of change of normalized capacitance. This is because the decrease of r increases the ACET microfluidic velocity, which causes more ctDNA to flow to and bind to the probe fixed on the electrode surface per unit time, resulting in a rapid change of normalized capacitance. Therefore, subsequent experiments were performed using chips with a size of 5 μm × 5 μm.

S.3. Electrochemical Responses at Different Voltages

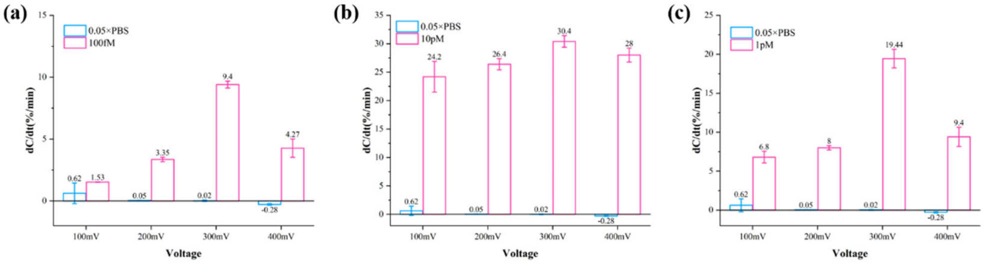

Figure S3. The rate of capacitance change of (**a**) 100 fM, (**b**) 1 pM, and (**c**) 10 pM ctDNA solutions at four voltages was detected using the method of Au–S bond fixation probes.

From Equation (1), the voltage amplitude V of the applied AC signal is also an important factor affecting the ACET microcurrent speed. The method of Au–S bond fixation

probes was used as an example, using a chip with a size of 5 μm × 5 μm, while the frequency was fixed at 20 kHz. The ctDNA solutions of 100 fM, 1 pM, and 10 pM were detected at four voltages of 100 mV, 200 mV, 300 mV and 400 mV, with 0.05 × PBS as blank control. The test results are shown in Figure S3. From the test results in Figure S3(a), Figure S3(b), and Figure S3(c), the normalized capacitance change rate of ctDNA solutions of all three concentrations increased with the increase of voltage when the applied voltage of AC signal increased from 100 mV to 300 mV. This is because the increase in voltage causes the ACET microfluidic velocity to increase, thus allowing more ctDNA to flow to and bind to the probe fixed on the electrode surface per unit time, resulting in a rapid change in normalized capacitance. However, when the voltage was further increased to 400 mV, the rate of change of the normalized capacitance decreased for the 3 concentrations of ctDNA solution compared to 300 mV. This may be since further increase in voltage leads to excessive ACET microfluidic velocity, which washes out the probe fixed on the electrode surface, causing less ctDNA to bind specifically to the probe per unit time and thus resulting in a lower rate of change of normalized capacitance. Therefore, the applied AC signal voltage amplitude was set to 300 mV for subsequent experiments.

S.4. Time Response of the Biosensor

Taking 10pM ctDNA as an example, the response of the biosensor reaches about 80% at 60 seconds (Figure S4), therefore we intercepted the 60s response value as the final response of the biosensor.

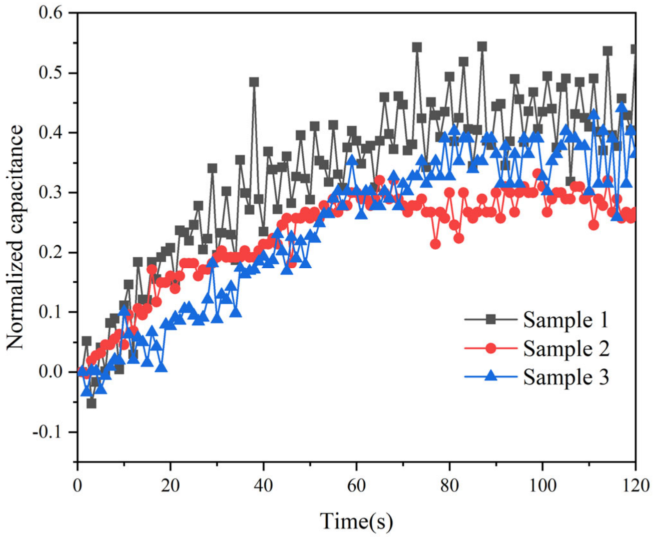

Figure S4. Time response characterization of ctDNA biosensors based on the ACEK effect.

S.5. Comparison of Different Electrochemical Biosensors

Table S1. Comparison of different electrochemical biosensors for PIK3CA ctDNA detection.

Hot spot mutation	Electrochemical Method	Linear Response Range	LOD[a]	Assay Time	Binding Method	Cost	Ref.
E542K	Normalized capacitance change rate	10 fM–10 pM	5.90 fM	1 min	Base complementary pairing	About $50	This work
		10 fM–10 pM	1.94 fM	1 min	Base complementary pairing	About $60	
E542K	SWV[b]	50–10000 fM	10 fM	30 min	Immune response	–	[2]
PIK3CA exon 9 mutation	EIS[c]	2–20 nM	0.65 nM	40 s	Base complementary pairing	–	[3]
E545K	DPV[d]	5 pM–0.5 nM	3 pM	30min	Base complementary pairing	–	[4]
E545K	DPV	10 fM–5 nM	8.3 fM	–	Base complementary pairing	–	[5]

[a] LOD: limit of detection.
[b] SWV: Square wave voltammetry.
[c] EIS: Electrochemical impedance spectroscopy.
[d] DPV: Differential pulse voltammetry.

References

1. Wu, J.; Lian, M.; Yang, K. Micropumping of Biofluids by Alternating Current Electrothermal Effects. Applied Physics Letters 2007, 90 (23), 234103.
2. Cai, C.; Guo, Z.; Cao, Y.; Zhang, W.; Chen, Y. A dual biomarker detection platform for quantitating circulating tumor DNA (ctDNA). Nanotheranostics 2018, 2 (1), 12-20.
3. Uygun, Z. O.; Yeniay, L.; Gïrgïn Sağın, F. CRISPR-dCas9 Powered Impedimetric Biosensor for Label-Free Detection of Circulating Tumor DNAs. Analytica Chimica Acta 2020, 1121, 35–41.
4. Huang, Y.; Tao, M.; Luo, S.; Zhang, Y.; Situ, B.; Ye, X.; Chen, P.; Jiang, X.; Wang, Q.; Zheng, L. A Novel Nest Hybridization Chain Reaction Based Electrochemical Assay for Sensitive Detection of Circulating Tumor DNA. Analytica Chimica Acta 2020, 1107, 40–47.
5. Luo, S.; Zhang, Y.; Huang, G.; Situ, B.; Ye, X.; Tao, M.; Huang, Y.; Li, B.; Jiang, X.; Wang, Q.; Zheng, L. An Enzyme-Free Amplification Strategy for Sensitive Assay of Circulating Tumor DNA Based on Wheel-like Catalytic Hairpin Assembly and Frame Hybridization Chain Reaction. Sensors and Actuators B: Chemical 2021, 338, 129857.
